# Validation of the confusion assessment method for intensive care

**DOI:** 10.1515/med-2026-1394

**Published:** 2026-03-02

**Authors:** Carla Cristina Nunes Teixeira Veiga, Paulo Alexandre Oliveira Marques, José Manuel Monteiro Dias, Amâncio António de Sousa Carvalho

**Affiliations:** Specialized Nurse in Critical Care, Local Health Unit of Trás-os-Montes and Alto Douro, Hospital of S. Pedro, Vila Real, Portugal; Nursing School, University of Porto, Porto, Portugal; RISE-Health, Porto, Portugal; Department of the School of Health of the University of Trás-os-Montes e Alto Douro, Vila Real, Portugal; CIEC – Research Centre on Child Studies, University of Minho, Braga, Portugal; Health School, University of Trás-os-Montes and Alto Douro, Quinta de Prados, Vila Real, Portugal

**Keywords:** delirium, intensive care units, risk assessment methodologies, mechanical ventilator, validation study, dimensional measurement accuracy

## Abstract

**Objectives:**

Delirium is a common but frequently underdiagnosed complication in adult intensive care units (ICU), associated with increased morbidity, mortality, and healthcare costs. Early detection using validated tools is essential. This study aim to translate, adapt, and validate the Confusion Assessment Method for Intensive Care Units flowsheet (CAM-ICU flowsheet) into European Portuguese.

**Methods:**

A methodological, cross-sectional, descriptive-correlational study was conducted in a northern Portuguese ICU. The CAM-ICU flowsheet was translated and back-translated, followed by expert validation. Forty-two adult ICU patients on invasive mechanical ventilation were assessed using the adapted tool by two trained observers. Diagnostic accuracy was evaluated using DSM-5 criteria as the gold standard. Statistical analysis included sensitivity, specificity, kappa coefficient, and ROC curves.

**Results:**

Delirium was detected in 57.1 % of patients. The CAM-ICU flowsheet in European Portuguese demonstrated high interobserver agreement (κ=0.952), excellent sensitivity (100 %), and specificity (81.8–86.3 %). ROC AUC ranged from 0.909 to 0.932. The inattentiveness feature was the core for the presence or absence of delirium.

**Conclusions:**

The CAM-ICU flowsheet in European Portuguese was validated to assess delirium in nonverbal ICU patients on mechanical ventilation through this study and demonstrated to be a reliable and valid tool.

## Introduction

At present, in light of scientific evidence, delirium rarely appears at the top of healthcare professionals’ priority lists. It often remains undetected because systematic monitoring is frequently not implemented in Intensive Care Units (ICUs) or in hospital services in general. The prevalence of delirium in ICUs ranges from 30 % to 80 % among critically ill adult patients and reaches up to 70 % in patients undergoing invasive mechanical ventilation (IMV), with an impact of up to 50 % in the older population. Exposure to delirium appears to be associated with increased morbidity and mortality, longer hospital length of stay, a higher risk of long-term cognitive decline, increased incidence of mental health disorders such as anxiety and depression, and the development of post-traumatic stress disorder symptoms after ICU or hospital discharge [1], [2], [3].

Recent studies conducted in critically ill patients with Acute Respiratory Distress Syndrome due to COVID-19 admitted to ICUs have reported a high incidence of delirium. This has been associated not only with critical illness and the potential direct viral effects on the central nervous system, but also with prolonged IMV, isolation, physical restraints, and extended exposure to sedative and analgesic drugs. These measures were often adopted to reduce the risk of ventilator circuit disconnection and/or self-extubation [1], 4].

Some scientific publications have highlighted the negative impact of delirium exposure on adult patients recovering from severe illness after hospitalization. For example, adult patients who experienced delirium were found to be less likely to recover their cardiac functional status compared with those who were not exposed to this syndrome [2].

Delirium is a multifactorial syndrome with a pathophysiological mechanism that remains incompletely understood. It represents a form of acute brain dysfunction and a neurocognitive disorder characterized by an altered and/or fluctuating level of consciousness, inattention, and disorganized thinking. It develops over a short period of time, ranging from hours to days, is potentially reversible, and results from an acute medical condition, exposure to toxins, intoxication or withdrawal from substances, or a combination of these factors. During delirium, patients experience impaired ability to receive, process, store, and retrieve information [5], 6].

Clinically, delirium presents with different psychomotor manifestations and is classified into subtypes: hyperactive (agitation and restlessness), hypoactive (lethargy and reduced motor activity), and mixed (alternating lethargy and agitation throughout the day). The mixed form is often referred to as “sundowning syndrome,” as agitation typically worsens in the late afternoon or evening. Over the past eight years, reviews focusing on delirium subtypes in adult ICU patients have consistently shown that hypoactive and mixed delirium are the most common presentations [5], 7]. An exception has been reported in patients with COVID-19, in whom studies, although subject to some bias, tend to demonstrate a slight shift towards hyperactive and mixed subtypes [4], 8].

The *Clinical Practice Guidelines for the Prevention and Management of Pain, Agitation/Sedation, Delirium, Immobility, and Sleep Disruption* (PADIS guidelines) [9], which reinforced the international paradigm shift initiated by the publication of the PAD guidelines in 2013 [10], highlighted the importance of evidence-based prevention and management of pain, agitation/sedation, delirium, immobility, and sleep disruption in adult ICU patients. With regard to delirium, there remains a lack of large, randomized or cohort, multicentre, and multinational studies capable of establishing robust evidence for direct associations between predisposing and/or precipitating factors and the occurrence of this syndrome. Consequently, the PADIS guideline panel reached consensus that, at the time, strong evidence supported a positive association between delirium and the use of continuous benzodiazepine sedation and multiple blood transfusions in critically ill adult patients as precipitating risk factors. Non-modifiable predisposing factors for delirium in adult ICU patients include advanced age, dementia, prior coma, emergency surgery or pre-ICU trauma, and high severity scores within the first 24 h, such as APACHE and ASA. More recent studies have associated delirium development with age over 74 years, the presence of more than two organ dysfunctions, chronic obstructive pulmonary disease, frailty syndrome, pain, severe electrolyte disturbances, fever, poor sleep quality, and physical restraints [9], [11], [12], [13].

Critically ill adult patients who experience one or more episodes of delirium during ICU admission are exposed to an additional factor that negatively affects post-discharge quality of life. Beyond its impact on individual health, delirium also has significant economic consequences for patients, families, and healthcare systems. Recent international publications indicate that, among older adults with or without surgical diagnoses admitted to ICUs or general hospital wards, the cumulative healthcare costs associated with delirium are comparable to those of cardiovascular disease and diabetes and are accompanied by worse outcomes. These costs are directly related to prolonged hospital length of stay in patients who develop delirium. There also appears to be an additional increase in costs when delirium occurs in patients with pre-existing dementia. In Portugal, a study examining delirium in critically ill adult ICU patients and its associated economic burden found that patients diagnosed with delirium incurred, on average, an additional cost of €2006.70 per patient by the end of ICU admission [14], [15], [16], [17], [18].

Several strategies exist to prevent or mitigate the impact of delirium. Among the most important is systematic monitoring using validated assessment tools adapted to ICU settings in different countries. This approach aims to enable early identification of delirium and is strongly emphasized in the 2018 guidelines, which classify daily delirium assessment in critically ill adults as Good Practice [9].

Early detection of delirium allows timely initiation of appropriate treatment and has been associated with prognoses similar to those of critically ill adult patients who do not develop delirium. Among the most recommended bedside assessment instruments for adult ICU patients are the *Confusion Assessment Method for the Intensive Care Unit* (CAM-ICU) and the CAM-ICU flowsheet. These tools can be applied by multidisciplinary team members without specialized training in mental health or psychiatry and demonstrate high sensitivity and specificity for diagnosing the presence or absence of delirium [9], 19], 20].

The motivation for delirium research and screening in the ICU setting began in 2009 during professional practice as a nurse providing direct care to critically ill adult patients in an intensive care service. Frequent situations were observed, such as difficulties in ventilator weaning in patients receiving IMV at the time of progressive withdrawal of advanced life support due to overall improvement in organ dysfunction. Conversely, there were also cases requiring escalation of ventilatory support due to worsening respiratory failure, often associated with sudden-onset severe confusional states within the first 24–48 h (These episodes included disorientation in time, space, and self-awareness). In clinical practice, this phenomenon was not generally regarded as clinically relevant by the multidisciplinary team. When it did receive attention, it was mainly in cases of hyperactive delirium, the most visible subtype and the one that posed greater risks to patient and staff safety. However, many patients appeared calm, provided simple and apparently appropriate responses, and did not display hyperactive or aggressive behavior. Despite this, they frequently removed medical devices or attempted to leave the bed, increasing the risk of falls due to acquired muscle weakness, among other adverse events. Based on nearly two decades of empirical observation in ICU care in Portugal, interest in delirium has increased and has become the focus of multiple academic theses, dissertations, and scientific publications, including review articles. These efforts have largely emphasized prevention strategies aligned with the PADIS guidelines. Nevertheless, real-world practice within intensive care services, as well as observations from scientific and professional exchanges between ICUs, indicate that adherence by multidisciplinary teams to delirium assessment remains below the level recommended by international guidelines over the past eight to nine years. The instrument in use, namely the CAM-ICU flowsheet in Brazilian Portuguese, has not been validated for the specific characteristics of the Portuguese population, which compromises the reliability of delirium assessment.

Within this context, investigating delirium and promoting its systematic screening using a validated ICU-specific tool became not only necessary but essential to support appropriate delirium assessment in critically ill adult patients.

At the time this study was conducted, no validation of the CAM-ICU flowsheet in European Portuguese was available. This gap justified the need to validate the CAM-ICU flowsheet for European Portuguese, which this original manuscript aims to address.

Accordingly, this study was conducted with the overall objective of translate, adapt and validate the CAM-ICU flowsheet into European Portuguese, in critically ill adult patients admitted to an Intensive Care Medicine Service (ICMS), in the northern interior of Portugal.

## Methods

This is a methodological, descriptive–correlational, cross-sectional study with a quantitative approach [21].

### Population and sample

The group of individuals with shared characteristics for this study was drawn from a population admitted to an ICMS of a hospital centre located in the northern interior region of Portugal. The CAM-ICU flowsheet is designed for application in critically ill adult patients admitted to the ICU, with or without the presence of an endotracheal tube or tracheostomy (ETT). In this study, only patients with an ETT who were unable to communicate verbally were included, given the additional challenge of identifying delirium using the aforementioned scale.

The inclusion criteria were defined as follows: (i) patients aged 18 years or older; (ii) absence of intellectual disability; (iii) absence of visual impairment; (iv) presence of an ETT or tracheostomy; (v) undergoing IMV; and (vi) with or without analgesia and/or sedation. Between 1 March 2016 and 31 August 2016, 386 patients were admitted to the ICMS. After applying the inclusion criteria, a population of 129 critically ill adult patients was identified.

The exclusion criteria were: (i) patients with a Richmond Agitation–Sedation Scale (RASS) score ≤ −3 at the time of observation [22], as RASS is used to assess the level of consciousness required for CAM-ICU flowsheet application and can be applied to both sedated and non-sedated patients [5]; (ii) IMV duration <48 h; (iii) limitation of therapeutic effort; and (iv) absence from the ICMS at 48 h, including patients transferred to other ICUs, those diagnosed with brain death, or deceased patients. After applying these criteria, the final sample consisted of 42 patients (32.6 % of the population). Specifically, 25 patients were excluded due to RASS ≤ −3, 31 due to IMV <48 h, 19 due to limitation of therapeutic effort, and 12 due to absence from the ICMS at 48 h. No formula was used to calculate the minimum sample size.

This was a non-probabilistic convenience sample, as participant selection followed a set of known population characteristics that were easily accessible (right place and right time) and met the exclusion criteria. Sampling was consecutive throughout the data collection period.

### Data collection instrument

A data collection form was developed to gather primary and secondary data, incorporating the CAM-ICU flowsheet translated and adapted into European Portuguese for subsequent validation [21].

The sequential procedures for translation and cultural adaptation of the CAM-ICU flowsheet into European Portuguese were based on the version published on the icudelirium.org website, which represents a restructuring of the CAM-ICU worksheet by Ely et al. [5], 23]. These procedures included: (i) prior authorisation requested by email from the original scale’s lead author for its use for the stated purposes, which was granted; (ii) translation of the scale from English into Portuguese with the support of an expert in mental health and psychiatric nursing, who verified terminological correspondence between the original and translated versions. During this process, a second set of letters was added to assess Feature 2 (Inattention), introducing an alternative option suggested by the original authors to improve fidelity. This resulted in a provisional European Portuguese version of the CAM-ICU flowsheet; (iii) back-translation into English by an independent certified bilingual expert who had no contact with the original version, thus avoiding translation bias. The back-translated version was then compared with the original English scale in the presence of the principal investigator, the bilingual expert, and the mental health specialist.

As no conceptual discrepancies requiring substantial changes were identified between the two versions, the provisional European Portuguese CAM-ICU flowsheet was finalized and incorporated into the data collection instrument developed for this validation study. The assessment method using this new flowsheet followed the original application steps and sequential evaluation of features.

(i) First step: the level of consciousness was assessed using the RASS. When the score was greater than −3, assessment proceeded to the second step, which involved evaluation of the content of consciousness using the CAM-ICU flowsheet.
(ii) Second step: the CAM-ICU flowsheet consists of four features to be assessed, any of which may determine the presence or absence of delirium.

The following procedures were adopted in the second step of scale application.

(i) **Feature 1 – Acute onset or fluctuating course**: assessed through either clinical records and/or information from family members regarding the patient’s mental status prior to the acute illness leading to ICU admission, or evidence of mental status fluctuation within the previous 24 h, obtained from nursing clinical records. If neither condition was present, assessment was terminated and delirium was considered absent. Otherwise, evaluation continued to the next feature.
(ii) **Feature 2 – Inattention**: assessed through standardized tests designed to infer the ability to receive, process, store, and retrieve information. The assessment sequence included an initial letter test (two sets of ten standardized alphabet letters from the original scale, allowing alternation across repeated assessments). If results were inconclusive, a visual test using images was applied (two sets of images depicting animals and objects, allowing alternation across assessments). These images were printed on laminated paper in standardized size and color as defined by the original authors. When the visual inattention test was used, scoring was based on this test alone: 0–2 errors indicated absence of delirium and assessment stopped, whereas more than 2 errors led to continuation to the next feature.
(iii) **Feature 3 – Altered level of consciousness (current RASS)**: assessed using the RASS score at the time of evaluation. A score different from 0 indicated delirium, whereas a score of 0 prompted assessment of the final feature.
(iv) **Feature 4 – Disorganized thinking**: consisted of two sets of four questions each, allowing alternation across repeated assessments, combined with two commands. These involved demonstrating a hand gesture with the fingers of one hand and asking the patient to imitate it, followed by a request to perform the same gesture with the opposite hand without demonstration. Commands were always performed sequentially after the questions to reduce the likelihood of random responses.

A data collection form incorporating primary and secondary data and the adapted European Portuguese version of the scale was developed. Approval for the study was obtained from the Ethics Committee of the Trás-os-Montes and Alto Douro Hospital Center (CHTMAD), in accordance with the ethical principles of the Declaration of Helsinki. A favorable opinion was issued in January 2016 (Opinion no. 5279).

### Data collection procedure

Data collection was carried out with the collaboration of two members of the ICMS multidisciplinary team, a nurse and a physician, working rotating shifts (morning, afternoon, and night). They were supervised by a mental health and psychiatric expert, who validated successive assessments based on DSM-5 criteria. During the six-month period from March to August 2016, data were collected whenever both trained observers were present simultaneously and patients meeting the study criteria were identified. Data collection always occurred with the presence of both observers and the principal investigator. The investigator was present during all data collection periods, whether during working hours or not, to ensure correct application of the data collection form by the observers who screened for the presence or absence of delirium. Observer training was conducted in the week preceding data collection and consisted of a demonstration of scale application by one of the investigators at the bedside of patients not included in the study. This training lasted approximately five hours and aimed to standardize application procedures. Delirium screening at the bedside was performed by one observer in the absence of the other for each patient. The results of successive assessments were subsequently reviewed with the mental health and psychiatric expert, in accordance with the assumption that higher inter-observer agreement reflects greater consistency in scale validation [21].

Primary data collected using the form were complemented with secondary data obtained through review of patients’ clinical records during the same period.

### Data processing and analysis

For statistical analysis, a database was created using the Statistical Package for the Social Sciences (SPSS) version 25.0. MedCalc Statistical Software version 18.5 (MedCalc Software bvba, Ostend, Belgium; http://www.medcalc.org; 2018) was additionally used for the construction of selected graphs. A type I error (α) of 0.05 or a 95 % confidence interval (CI) was considered.

Data analysis included descriptive and inferential statistics [24]. Descriptive statistics comprised calculation of absolute and relative frequencies for all categorical variables, as well as measures of central tendency, minimum, maximum, and standard deviation for ratio-level variables.

For validation of the European Portuguese CAM-ICU flowsheet, sensitivity, specificity, positive predictive value (PPV), negative predictive value (NPV), and likelihood ratios (LRs; positive LR and negative LR) were calculated to assess diagnostic test performance. Cohen’s Kappa coefficient (κ) was also used to evaluate inter-observer agreement between Observer A and Observer B by comparing observed agreement with expected agreement. Agreement strength was classified into five levels: poor, fair, moderate, good, and very good [24]. Receiver Operating Characteristic (ROC) curves were constructed to examine variations in sensitivity and specificity across different cut-off points (ranging from 0 to 1). The area under the ROC curve (AUC) was analyzed to assess the scale’s ability to discriminate individuals with the characteristic of interest. An AUC value closer to 1 indicates greater discriminatory power. Based on AUC and likelihood ratios, diagnostic test quality was classified as Good, Very Good, or Excellent [24], 25].

## Results

Of the total patient sample (n=42), most were male (69 %). The mean age was 66.9 ± 11.36 years old (minimum 44, maximum 87 years), and the majority belonged to the age group of 65 years or older (64.3 %).

A predominance of medical diagnostic categories was observed in 57.1 % of patients. The most prevalent pre-existing risk factor was arterial hypertension (54.8 %). Criteria for sepsis or septic shock associated with the admission diagnosis were present in 81.0 % (n=34) of patients. Regarding severity indices at 24 h after admission to the ICMS, organ dysfunction indices (at admission and discharge), and nursing workload related to direct patient care, the following mean values were observed: Acute Physiology and Chronic Health Evaluation II (APACHE II), 20.45 ± 7.92 (minimum 3, maximum 36); Simplified Acute Physiology Score II (SAPS II), 46.43 ± 12.41 (minimum 23, maximum 79); Sequential Organ Failure Assessment (SOFA) at admission, 7.50 ± 3.41 (minimum 1, maximum 15); SOFA at ICMS discharge, 2.64 ± 2.26 (minimum 0, maximum 13); and Therapeutic Intervention Scoring System 28 (TISS-28), 37.10 ± 3.83 (minimum 30, maximum 46). Continuous intravenous sedative infusion was administered to 64.3 % (n=27) of patients, with propofol 2 % being the most frequently used agent (45.2 %). Analgesic medication was administered to 97.6 % (n=41) of patients, either as continuous intravenous infusion and/or bolus dosing. The most commonly prescribed analgesics were morphine (38.1 %, n=16 patients) and tramadol (38.1 %, n=16 patients), both administered as continuous infusion.

The mean length of stay in the ICMS was 14.38 ± 8.47 days (minimum 3, maximum 35 days). The mean duration of IMV was 10.31 ± 6.13 days (minimum 2, maximum 23 days). Overall, 9.5 % (n=4) of patients died during their ICMS stay.

### Assessment of the presence of delirium

Delirium was identified in 57.1 % (n=24) of the patients. In most cases, scale application occurred at RASS levels of −1 (28.6 %, n=12 patients) and −2 (23.8 %, n=10 patients). Among the four features assessed by the scale, Feature 2 (inattention) was the core determinant for the presence or absence of delirium in 45.2 % (n=19) of the sample.

### Inferential analysis of delirium in relation to sociodemographic and clinical characteristics

No statistically significant differences were observed in the prevalence of delirium according to sex or age group (χ^2^: p≥0.333 and χ^2^: p≥0.347, respectively).

Inferential analysis between clinical characterization variables and the presence of delirium revealed the following findings.

– Statistically significant differences were observed between diagnostic categories (χ^2^: p<0.030). Delirium was more frequent among patients with medical and trauma diagnoses, with adjusted residuals of +1.4 and +1.1 cases above expected values, respectively. In contrast, the elective surgery diagnostic category showed an adjusted residual of −2.8 cases below the expected value, with no cases of delirium identified (Table 1).
– The presence of delirium was not associated with pre-existing risk factors in the sample (χ^2^: p≥1.000), nor with exposure to sedative medication (χ^2^: p≥1.000) (Table 1).
– Statistically significant differences were observed between patients with and without criteria for sepsis or septic shock and the presence of delirium (χ^2^: p<0.013). Patients meeting these criteria had an adjusted residual of +2.8 cases above the expected value and a higher prevalence of delirium compared with those without sepsis or septic shock ( vs. 12.5 %) (Table 1).

Among patients with delirium, analysis of age, length of stay in the service, duration of invasive mechanical ventilation, severity indices at 24 h (APACHE II and SAPS II), organ dysfunction indices (SOFA at admission and discharge), and nursing workload related to direct patient care (TISS-28) showed that statistically significant differences were observed only between the presence of delirium and higher APACHE II scores at 24 h, as shown in Table 2.

**Table 1:** Inferential analysis between the presence of delirium and the diagnostic category, previous risk factors, sedative medication and presence of sepsis/septic shock criteria of the patients in the sample (n=42).

Variables	Presence of delirium				χ^2^ test	df	p-Value
	Yes (24)		No (18)				
	n	%	n	%			
Diagnostic category							
Medical (24)	16	66.7	8	33.3	8.789	3	0.030
Urgent surgery (8)	4	50.0	4	50.0			
Scheduled surgery (5)	0	0.0	5	100.0			
Trauma (5)	4	80.0	1	20.0			
Previous risk factors							
Yes (37)	21	56.8	16	43.2	0.019	1	1.000
No (5)	3	60.0	2	40.0			
Sedative medication							
Yes (27)	15	55.6	12	44.4	0.078	1	1.000
No (15)	9	60.0	6	40.0			
Sepsis/septic shock							
Yes (34)	23	67.6	11	32.4	8.042	1	0.013
No (8)	1	12.5	7	87.5			

Legend: df, degree of freedom; n, absolute frequency; p, probability of significance %, relative frequency; χ^2^, Chi-square.

**Table 2:** Multivariate analysis between the presence of delirium and the continuous independent variables of age, length of stay in the service, days of VMI, APACHE II, SAPS II, SOFA (admission and discharge), TISS28 of the patients in the sample (n=42).

Variables	Presence of delirium									
	Yes (24)			No (18)			*Wald* Test	df	p-Value	Exp (B)
	Me ± sd	Min	Max	me ± sd	min	Max				
Age	67.33 ± 11.627	45	84	66.33 ± 11.324	44	87	0.062	1	0.803	0.990
Delay in service	14.79 ± 7.690	3	35	13.83 ± 9.624	3	32	0.579	1	0.447	1.116
VMI days	10.33 ± 6.873	2	23	10.28 ± 6.649	2	22	0.842	1	0.359	0.839
APACHE II	23.21 ± 7.824	10	36	16.78 ± 6.612	3	30	6.090	1	0.014	1.121
SAPS II	50.58 ± 13.045	23	79	40.89 ± 9.171	25	58	0.147	1	0.701	1.023
SOFA										
Admission Discharge	8.63 ± 2.716 2.67 ± 2.382	3 0	15 13	6.00 ± 3.726 2.61 ± 1.819	1 1	12 9	1.633 0.856	1 1	0.201 0.355	1.211 0.842
TISS 28	37.75 ± 4.225	30	46	36.22 ± 3.154	31	42	0.202	1	0.653	1.050

Legend: df, degrees of freedom; Exp(B), exponential of the model coefficients/odds ratio: probability of the patient developing delirium; me ± sd, mean and standard deviation; min, minimum; max, maximum; p, probability of significance.

In summary, inferential analysis between the different variables (categorical and continuous) and the presence of delirium demonstrated a dependent relationship between delirium and diagnostic category, the presence of sepsis or septic shock criteria, and APACHE II scores.

### Validation of the CAM-ICU flowsheet in European Portuguese

Validation of the European Portuguese version of the CAM-ICU flowsheet was conducted as follows: (i) analysis of data obtained from scale application by two observers (A and B) to determine the presence or absence of delirium; and (ii) verification against a gold standard established by a mental health and psychiatric expert according to DSM-5 criteria.

Within the study sample, Observer A identified delirium in 57.1 % (n=24) of patients, while Observer B identified delirium in 54.8 % (n=23). According to DSM-5 criteria, delirium was present in 47.6 % (n=20) of patients.

Regarding delirium classification, inter-observer agreement between Observers A and B was classified as very good and statistically significant (κ=0.952; p<0.001) (Table 3).

**Table 3:** Analysis of interobserver agreement A and B with the application of the CAM-ICU flowsheet in European Portuguese in the patients in the sample (n=42).

Variables	Presence of delirium (observer A)				*k* test value	p-Value
	Yes (24)		No (18)			
	n	%	n	%		
Presence of delirium (observer B)						
Yes (23)	23	100.0	0	0.0	0.952	0.000
No (19)	18	94.7	1	5.3		

Legend: n, absolute frequency; %, relative frequency; *k*, Cohen’s Kappa; p, probability of significance.

A very good and statistically significant level of agreement was also observed between the observers and the expert assessment, in accordance with DSM-5 criteria, for the evaluation of the presence or absence of delirium using the scale under study. Agreement was observed for Observer A (κ=0.811; p<0.001) and Observer B (κ=0.858; p<0.001) (Table 4).

**Table 4:** Analysis of agreement between observations A and B with the application of the CAM-ICU flowsheet in European Portuguese and presence/absence of delirium according to DSM 5 criteria, in the patients in the sample (n=42).

Variables	Presence of delirium (expert based on DSM 5)				*k* test value	p-Value
	Yes (20)		No (22)			
	n	%	n	%		

**Presence of delirium (observer A)**						

Yes (24)	20	83.3	4	16.7	0.811	0.000
No (18)	0	0.0	18	100.0		

**Presence of delirium (Observer B)**						

Yes (23)	20	87.0	3	13.0	0.858	0.000
No (19)	0	0.0	19	100.0		

Legend: n, absolute frequency; %, relative frequency; *k*, Cohen’s Kappa; p, probability of significance.

Application of the CAM-ICU flowsheet requires adherence to two sequential steps: assessment of the level of consciousness (first step) and assessment of the content of consciousness (second step). Agreement between observers (A and B) was observed for the level of consciousness among patients in the sample, namely at RASS -2 in 87.5 % (n=7), RASS -1 in 70.0 % (n=7), RASS 0 in 81.8 % (n=9), and RASS +1 in 63.6 % (n=7), indicating good agreement strength (κ=0.661; p<0.001). Regarding application of the scale itself, among the four features assessed to determine the presence or absence of delirium, the highest levels of agreement between observers (A and B) were observed for Feature 2 (Inattention), with 85.0 % agreement (n=17 concordant cases), and Feature 3 (Altered level of consciousness – current RASS), with 92.9 % agreement (n=13 concordant cases). These results indicate very good agreement strength (κ=0.817; p<0.001).

When applied by Observer A and compared against DSM-5 criteria as the gold standard, the European Portuguese CAM-ICU flowsheet achieved a sensitivity of 100 % and a specificity of 81.8 %. This reflected excellent discrimination between the presence (sensitivity) and absence (specificity) of delirium, as demonstrated by the area under the ROC curve (AUC=0.909; p<0.0001). Similarly, application of the scale by Observer B, using DSM-5 criteria as the gold standard, resulted in a sensitivity of 100 % and a specificity of 86.3 %, with excellent discriminatory ability confirmed by the ROC curve analysis (AUC=0.932; p<0.0001).

Accordingly, when DSM-5 criteria were adopted as the gold standard for identifying the presence or absence of delirium in the sample (47.6 % with delirium and 52.4 % without delirium), the European Portuguese CAM-ICU demonstrated good predictive performance. Sensitivity and specificity exceeded 80 %, with positive predictive values (PPV) of 83.3 % for Observer A and 86.3 % for Observer B. Negative predictive values (NPV) were 100 % for both observers. Diagnostic test quality, assessed using AUC values and likelihood ratios (positive and negative), was classified as Good for Observer A (AUC=0.909; +LR=5.50; −LR=0.00; p<0.0001) and Very Good for Observer B (AUC=0.932; +LR=7.33; −LR=0.00; p<0.0001) (Table 5).

**Table 5:** Validation of the CAM-ICU flowsheet in European Portuguese using the DSM 5 criteria as the gold standard.

Variables	Sensitivity (IC 95 %)	Specificity (IC 95 %)	PPV	NPV	+LR	-LR	*AUC (CI 95 %)*	p-Value
Observers								
A	100.0 % (83.2–100.0)	81.8 % (59.7–94.8)	83.3 %	100 %	5.50	0.00	0.909 (0.779–0.976)	0.0001
B	100.0 % (83.2–100.0)	86.3 % (65.1–97.1)	86.9 %	100 %	7.33	0.00	0.932 (0.810–0.986)	0.0001

Legend: AUC, (area under the ROC curve); CI, confidence interval; +LR, (likelihood ratios) positive likelihood ratio; −LR, (likelihood ratios) negative likelihood ratio; NPV, negative predictive value; PPV, positive predictive value; p, probability of significance; %, relative frequency.

## Discussion

Traditionally, the identification of delirium relied exclusively on the application of criteria defined in the *Diagnostic and Statistical Manual of Mental Disorders* (DSM) and could only be performed by healthcare professionals with specific training in psychiatry. This represented a major barrier to delirium screening in ICU settings, particularly because multidisciplinary teams often lack professionals with psychiatric expertise. The development, adaptation, and validation of the CAM-ICU assessment tool in 2001 addressed this gap by enabling ICU professionals without formal training in mental health or psychiatry to identify the presence or absence of delirium through structured multidisciplinary training based on the CAM-ICU manual. This approach also allowed identification of the delirium subtypes most commonly observed in ICU settings [23], 26].

Over a 12-year period, the scientific community focused on critically ill adult patients in ICUs analysed international evidence on best practices related to pain, agitation, and delirium. This work led to the formation of a task force that published guidelines in 2013 addressing the assessment, monitoring, prevention, and treatment of this ICU triad. This represented the first major paradigm shift, highlighting the urgency of monitoring these phenomena using validated assessment scales adapted to the characteristics of adult ICU patients in each country. Among the instruments that achieved the greatest consensus for bedside delirium assessment was the CAM-ICU, which demonstrated high inter-rater reliability among nurses and physicians, as well as high sensitivity and specificity when compared with diagnostic criteria established by the American Psychiatric Association. These findings are consistent with the results of the present study. This consensus was further reinforced in the expanded 2018 PADIS guidelines [9], 10].

Accordingly, the present study focused on the translation, cultural adaptation, and validation of the CAM-ICU flowsheet in a sample of 42 critically ill adult patients. The sample predominantly included patients aged 65 years or older, with a mean age of 66.9 ± 11.36 years, and was mostly male (69 %). The most prevalent pre-existing risk factor was arterial hypertension (54.8 %). Most patients belonged to the medical diagnostic category (57.1 %), with 81.0 % meeting criteria for sepsis or septic shock. Severity indices at 24 h after ICU admission indicated moderate to severe illness (APACHE II: 20.45 ± 7.93; SAPS II: 46.43 ± 12.41), with substantial multiorgan dysfunction at admission (SOFA: 7.50 ± 3.41) and high nursing workload for direct patient care (TISS-28: 37.10 ± 3.84).

The literature on delirium in ICU settings is heterogeneous and includes adult critically ill populations with and without IMV. Many studies focus primarily on comparative characteristics between patients with and without delirium. Analysis of these studies indicates that the subgroup of mechanically ventilated patients tends to be aged 65 years or older, predominantly male, with arterial hypertension as a common pre-existing risk factor, mostly belonging to the medical diagnostic category, and presenting moderate to severe APACHE II scores at ICU admission. These findings are consistent with the results of the present study [1], 10], 27], 28].

At the time of data collection, continuous intravenous sedation was administered to 64.3 % of patients, with propofol 2 % being the most frequently used sedative. There was also a growing trend towards the introduction of alpha-2 adrenergic agonists, such as dexmedetomidine, either alone or in combination, as analgo-sedative agents. Analgesia was administered to 97.6 % of patients, primarily using morphine or tramadol. Although multimodal analgesia and fentanyl-based infusion, as recommended by the PADIS guidelines, were still limited, the near-universal provision of analgesia reflects early awareness within the multidisciplinary team of the importance of patient comfort and pain control. Sedation was primarily used to facilitate adaptation to IMV or to manage agitation during ventilator weaning. These practices suggest early adoption of evidence-based conscious sedation strategies, which promote patient interaction and reduce the risk of delirium, with positive effects on outcomes [9], 12], 29].

In this sample, delirium was identified in 57.1 % of patients using the European Portuguese version of the CAM-ICU flowsheet. Scale application most frequently occurred at RASS levels of −1 and −2, and Feature 2 (inattention) was the core determinant for delirium identification in 45.2 % of cases. These findings are consistent with hypoactive delirium, which is common in mechanically ventilated patients receiving light sedation [29].

Inferential analysis revealed a dependent relationship between delirium and diagnostic category, presence of sepsis or septic shock criteria, and higher APACHE II scores. Patients who developed delirium were more frequently from medical or trauma diagnostic categories, met sepsis or septic shock criteria, and presented higher illness severity. These associations are consistent with the PADIS guidelines and with findings from other recent studies and reviews [7], 9], 13].

In contrast, delirium occurrence was independent of age, sex, pre-existing risk factors, sedative exposure, duration of IMV, and length of ICU stay, in line with current evidence [9], 30].

Although patients with delirium tended to have higher SAPS II scores, greater organ dysfunction at admission, increased nursing workload, and longer ICU stays, multivariate analysis did not confirm these trends, likely due to the small sample size. Overall, the sample consisted of critically ill patients with an estimated hospital mortality risk of 25–50 %, significant multiorgan dysfunction, and high nursing care demands, corresponding predominantly to TISS-28 class III patients, who are considered severely ill and hemodynamically unstable [31], 32].

Based on the philosophy for validating this scale, the results of the validation of the CAM-ICU flowsheet in European Portuguese were based on observations by two professionals from the ICMS team, a nurse and a doctor (Observer A and Observer B), contrasting these results with the gold standard of applying the DSM 5 criteria, by the expert in mental health and psychiatry [6], 23].

Thus, with the application of the scale, the presence of delirium occurred in 57.1 % of the patients in the sample, according to Observer A, and in 54.8 %, according to Observer B. Applying the DSM 5 criteria, the presence of the event occurred in 47.6 % of the patients in the study. Based on the classification of Pestana and Gageiro [25], the strength of agreement of Cohen’s Kappa (k) of interobservers A and B was very good and significant, the same classification occurring when the different observations, by observers A and B, were compared with the assessment by the expert in mental health and psychiatry for the presence of delirium.

Inter-observer agreement in this study was comparable to validation studies of the CAM-ICU in Hindi (κ=0.944; p<0.001), Chinese (κ=0.920; p<0.001), Tibetan (κ=0.910; p<0.001), and Chilean Spanish (κ=0.910; 95 % CI: 0.860–0.960) [26], [33], [34], [35]. Agreement between observers and expert assessment was similar to that reported in the Thai version (κ=0.810; p<0.001) and higher than that observed in the Greek version (κ=0.750; 95 % CI: 0.590–0.910) [36], 37].

It was found that the RASS (consciousness) levels most frequently tracked in the study sample by the two observers (A and B), which demonstrated the safety of the moment of application of the scale itself, were the levels of: ‘sedation’ (drowsiness and mild sedation – hypoactivity) ‘restless’ (anxious, but without aggressive and/or vigorous movements). This observation by the two professionals resulted in good interobserver agreement [22], 25].

The minimum level of consciousness for applying the CAM-ICU flowsheet, for this study, was RASS greater than −3. According to the training manual developed by Dr. Ely and available in its latest revised version of 2016 [5], the minimum cutoff is RASS -3. However, the author notes that, given the different international studies that have emerged since the publication of the CAM-ICU in 2001, with subsequent translations and validations for other languages/countries, he accepts that a RASS of −2 or −1 be adopted as the minimum lower limit (sedated or non-sedated patients) of ‘consciousness’ to apply the CAM-ICU (verification of the content of consciousness). The justification is simple: Dr. Ely advocates for safety in the results obtained from applying the scale to critically ill adult patients with a minimum RASS score of −3, meaning patients who show eye movement or opening to the voice, but without directing their gaze with eye contact to the person verbally stimulating them, and not always obtaining simple motor responses such as a handshake and/or a ‘yes’ or ‘no’ (with or without nodding). Other authors, as in the present study, based on their experience in clinical practice, consider that they have greater safety in the results obtained from applying the scale to patients when they adopt minimum ‘levels of consciousness’ of RASS −2 or −1, reiterating that some of the advantages are the existence of minimal eye contact and the presence of a simple response (‘yes’/‘no’ and/or handshake) to the professional and to the questions asked during the application of the scale [5], [38], [39], [40], [41].

As a pedagogical process, in the application of the four characteristics that make up the CAM-ICU flowsheet scale, the observers assessed the strength of interobserver agreement (A and B) for the presence or absence of the phenomenon in the patients in the sample. This was found to be very good in the choice of the attention test (visual or auditory), and the result of Feature 2 coincided in 85.0 % (17 patients coinciding between the two observers). Equal strength of agreement was observed when Feature 3 (Altered level of consciousness – current RASS) was indicated as the decisive moment for the presence of delirium.

In the studies consulted on the application of the CAM-ICU, and specifically in the flowsheet, at least one was found in which the good interobserver agreement resembled that of this study, regarding the result of the application of Feature 3 [42]. An agreement greater than 80 % interobserver, for Feature 2 (Inattention) and for Feature 3, highlights the importance of formal training (theoretical-practical and/or simulation), structured and in the context of clinical practice for the multidisciplinary team, for the management of this syndrome, in which the first approach must always be the correct application of the assessment scale, as was the case with the ICMS where the present study was carried out, an assertion by some of the authors consulted [36], [42], [43], [44].

The CAM-ICU and the CAM-ICU flowsheet have been the most internationally validated scales for application in critically ill adult ICU patients, with or without sedation, and with or without orotracheal intubation/tracheostomy, as mentioned in the table of this study. The presence of IMV, via orotracheal intubation or tracheostomy, makes the application of this type of scale at the bedside challenging, as this subpopulation has the most difficulty communicating verbally, and up to 70 % of these critically ill adult patients are likely to experience delirium. For this reason, this study aimed to validate the CAM-ICU flowsheet in European Portuguese in the subgroup of critically ill adult patients undergoing IMV. This validation could be generalized to the population of critically ill adult patients in the ICU, the context of this study, and the study could be replicated in other similar ICUs in Portugal.

Using DSM-5 criteria as the gold standard, the European Portuguese CAM-ICU flowsheet demonstrated excellent discrimination between delirium presence and absence, supported by ROC curve analysis. Sensitivity and specificity exceeded 80 %, with a NPV of 100 %. Diagnostic quality was classified as good for Observer A and very good for Observer B [24], 25].

In a search of similar validation studies in the international setting, in populations of critically ill adult patients in the ICU, mostly with samples of patients on and off mechanical ventilation, using DSM criteria as the gold standard, the updated French version from 2014 shows a sensitivity and specificity profile similar to that of this study, where the NPV of the French version was lower (91 % vs. 100 %), although in the French study the PPV is higher (100 % vs. 83.3–86.3 %) [44]. In the Tibetan version, the accuracy of the scale validation is consistent with the scale under study, with sensitivity and specificity results above 80 % and PPV between 83 and 89 %, but lower NPV (92–94 %) [34]. Prior to the validation process of the scale in the European Portuguese version, the Brazilian Portuguese, Chinese, and Thai versions of the CAM-ICU also reported generally high sensitivities and specificities, but with variability in PPV and NPV values, none reaching an NPV of 100 %, as in this study [20], 33], 36]. In the studies consulted, it was found that the validation of the CAM-ICU in critically ill patient populations in various languages, when compared to the DSM criteria as a reference standard, generally presents good validity and reliability, however, in none of the studies was there an NPV of 100 %, as in the present validation of the scale.

With the application of the CAM-ICU flowsheet in European Portuguese by the two Observers, all patients in the sample who experienced delirium were flagged, obtaining a model with good predictive capabilities (sensitivity and specificity greater than 80 %) [24], which supports the importance of training history in this area in the ICMS targeted by the study. The reference point for the diagnosis of the presence of delirium was the DSM 5 applied by the Specialist, and all cases flagged by him were flagged by the observers. However, in the absence of the phenomenon, the cases flagged by the expert were 22 patients in the sample, and by Observers A and B, 18 and 19 patients were flagged, respectively. These results can be justified by the fluctuating nature of delirium and some difficulty, of the scale in question, in signaling cases of subsyndromal delirium, especially when the conclusive result of the assessment of presence or absence of delirium is Feature 3, not applying to all characteristics, as suggested by some of the authors consulted [34], [45], [46], [47].

For a conclusive diagnosis of delirium, Features 1+2+3 or 4 must be present. Subsyndromal delirium is understood to be present when, in the application of the CAM-ICU to the critically ill adult patient, the patient manifests signs of cognitive dysfunction; that is, one or two of the evaluated characteristics are present, but do not meet all the necessary criteria for the presence of the syndrome. It is considered a transient phase of clinical interest because it can either evolve to normalization of mental status or deteriorate into delirium, particularly in patients undergoing mechanical ventilation. For some authors, the main difficulties lie in the lack of training on monitoring and evaluating delirium among multidisciplinary ICU teams. Others point to the assessment being done only once a day, which compromises the tracking of the fluctuating nature of the phenomenon and, in this case, increases the potential risk of never or almost never identifying a subsyndromal phase. For other authors, dichotomous scales such as the CAM-ICU will not be as specific for screening this underdiagnosis, a fact that has been refuted by what has been described previously [5], 47].

The results obtained in this study of translation and adaptation of the CAM-ICU flowsheet into European Portuguese confirm the validation of the scale for critically ill adult patients in an ICU, with good predictive capabilities for signaling the presence or absence of delirium. A NPV of 100 % stands out as a new finding, and no similar value was found among the studies consulted up to the time of writing this study.

The possible biases that may have affected this validation study were sample selection bias, small sample size bias, bias of delirium prevalence over the Kappa coefficient, observer bias, and temporal variation of delirium. These biases were partially mitigated through the rigorous definition of inclusion and exclusion criteria, the use of consecutive sampling over the time period during which data collection occurred, standardized training of observers, and the performance of independent assessments and comparison with the assessment performed using the DSM-5 criteria. In fact, we are aware that this is a small sample. As always, Gwet [48] reports that pilot and initial agreement validation studies often use 30–50 subjects, a range that the sample in the current study adheres to. This author also states that methodological studies suggest that samples with fewer than 30 to 50 paired observations allow for an accessible preliminary estimate of interobserver agreement.

Thus, the present study presents some limitations that should be considered in the interpretation of the results and conclusions. First, the small number of patients included, in relation to the total number of eligible patients, may introduce selection bias and limit the generalizability of the findings. Additionally, the relatively small sample size may affect the accuracy of the Kappa coefficient estimate. The distribution of delirium prevalence in the sample may also have influenced the Kappa value, given the known effect of prevalence on this measure of agreement. Because it is a fluctuating clinical picture, the temporal variability of delirium between assessments may have contributed to discrepancies between observers not attributable to instrument failures. Despite the prior training of the evaluators, the influence of observer bias cannot be completely excluded. However, the performance of independent assessments and the use of a standardized protocol sought to mitigate these factors. Thus, the results should be interpreted with caution, being applicable mainly to the population of critically ill patients assessable with the CAM-ICU scale.

This study was possible at the time because it involved the collaboration of the entire multidisciplinary team, especially two Observers from the service’s multidisciplinary team (a Nurse and a Doctor), who were aware of the importance of monitoring delirium in all admitted patients. The focus on the daily and systematic assessment of this syndrome (morning/afternoon/early evening), similar to the assessment of any other vital sign, is one of the initial pillars for the prevention and/or resolution of delirium in critically ill adult ICU patients, influencing the reduction of morbidity/mortality.

This intervention is only carried out correctly when validated instruments for these populations are used, in the respective native languages of each country. The introduction of the CAM-ICU flowsheet, validated in European Portuguese, will allow multidisciplinary teams to improve and ensure the effectiveness in identifying delirium in critically ill adult ICU patients. This effectiveness will have to be based on the training of these professionals, addressing the difficulties encountered, and on the construction of institutional norms, protocols, or algorithms that help standardize monitoring procedures for this syndrome. Furthermore, one of the quality criteria is the existence of this validated scale for the Portuguese context, as it is the one recommended for monitoring the affected indicator for critically ill patients by the Portuguese Society of Intensive Care. In addition, this version could be adopted nationally by other services similar to that of this study, thus representing an advantage and a benefit for the specific area of critically ill patients.

Future studies should prioritize larger, multicentre samples to improve reliability estimates and generalizability. Additional psychometric properties, including concurrent validity, test-retest reliability, and responsiveness, should also be evaluated. The CAM-ICU demonstrated adequate inter-observer agreement when applied by trained professionals, supporting its routine use for delirium screening in adult ICU patients. Early detection enables timely preventive and therapeutic interventions, with potential benefits for length of stay, complications, and functional outcomes.

The results of this study suggest that the CAM-ICU shows adequate interobserver agreement when applied by trained professionals, supporting its use as a systematic screening tool for delirium in adult patients admitted to the ICU. Early detection of delirium is fundamental for the timely implementation of preventive and therapeutic strategies, with potential impact on length of stay, associated complications, and functional prognosis. Routine use of the CAM-ICU can contribute to the standardization of mental status assessment, improve communication among healthcare professionals, and increase team awareness of delirium as a frequent and clinically relevant complication in intensive care.

Effective implementation requires ongoing training, standardized assessment schedules, protocol integration, and continuous monitoring of adherence and reliability. Key barriers in Portugal include high workload, time constraints, limited training, cultural under-recognition of delirium, limited electronic integration, and staff turnover. Facilitators include the simplicity of the scale, strong nursing engagement, clinical leadership, alignment with international recommendations, and inclusion in clinical protocols and audit processes.
